# Innovative Approaches to Assess Intermediate Cardiovascular Risk Subjects: A Review From Clinical to Metabolomics Strategies

**DOI:** 10.3389/fcvm.2021.788062

**Published:** 2021-12-22

**Authors:** Aline M. A. Martins, Mariana U. B. Paiva, Diego V. N. Paiva, Raphaela M. de Oliveira, Henrique L. Machado, Leonardo J. S. R. Alves, Carolina R. C. Picossi, Andréa T. Faccio, Marina F. M. Tavares, Coral Barbas, Viviane Z. R. Giraldez, Raul D. Santos, Guilherme U. Monte, Fernando A. Atik

**Affiliations:** ^1^Centre of Metabolomics and Bioanalysis (CEMBIO), San Pablo CEU University, Madrid, Spain; ^2^School of Medicine, University of Brasilia, Brasilia, Brazil; ^3^School of Medicine, University Center of Brasilia (UniCeub), Brasilia, Brazil; ^4^Center for Multiplatform Metabolomics Studies (CEMM), University of Sao Paulo, São Paulo, Brazil; ^5^Lipid Clinic, Heart Institute (InCor), University of Sao Paulo Medical School, São Paulo, Brazil; ^6^Department of Heart Transplant, Federal District Institute of Cardiology (ICDF), Brasilia, Brazil

**Keywords:** risk stratification, coronary artery disease (CAD), metabolomics, atherosclerosis, cardiovascular prevention

## Abstract

Current risk stratification strategies for coronary artery disease (CAD) have low predictive value in asymptomatic subjects classified as intermediate cardiovascular risk. This is relevant because not all coronary events occur in individuals with traditional multiple risk factors. Most importantly, the first manifestation of the disease may be either sudden cardiac death or acute coronary syndrome, after rupture and thrombosis of an unstable non-obstructive atherosclerotic plaque, which was previously silent. The inaccurate stratification using the current models may ultimately subject the individual to excessive or insufficient preventive therapies. A breakthrough in the comprehension of the molecular mechanisms governing the atherosclerosis pathology has driven many researches toward the necessity for a better risk stratification. In this Review, we discuss how metabolomics screening integrated with traditional risk assessments becomes a powerful approach to improve non-invasive CAD subclinical diagnostics. In addition, this Review highlights the findings of metabolomics studies performed by two relevant analytical platforms in current use–mass spectrometry (MS) hyphenated to separation techniques and nuclear magnetic resonance spectroscopy (NMR) –and evaluates critically the challenges for further clinical implementation of metabolomics data. We also discuss the modern understanding of the pathophysiology of atherosclerosis and the limitations of traditional analytical methods. Our aim is to show how discriminant metabolites originated from metabolomics approaches may become promising candidate molecules to aid intermediate risk patient stratification for cardiovascular events and how these tools could successfully meet the demands to translate cardiovascular metabolic biomarkers into clinical settings.

## Highlights

- Traditional stratification methods predict inaccurately the risk of cardiovascular events in intermediate risk asymptomatic subjects.
- A better understanding of the pathophysiology could provide accurate molecular signatures for subclinical atherosclerosis.
- Metabolomics couple with high performance analytical tools, as MS and NMR, would improve this knowledge.
- Clinical applications of metabolomics depend on its discriminant capability of individuals' reclassification.

## Introduction

Despite all improvements in prevention, diagnosis, and treatment, coronary artery disease (CAD) remains a leading cause of morbidity and mortality globally (1–5). Clearly further strategies are required to reduce the prevalence of this condition, which has implications both for the healthcare budget and, most importantly, the patient. Primary prevention has contributed substantially to the reduction in mortality rates and, even though most atherosclerotic cardiovascular disease events are avoidable through primordial prevention and control of traditional cardiovascular risk factors (6), there is no single risk calculator appropriate for all patients (7–10).

Because acute coronary syndromes (ACS) are often the first manifestations of CAD in previously asymptomatic individuals, there is an ongoing debate regarding how to improve the current tools used in clinical practice to predict the risk of a future acute myocardial infarction (MI) (11, 12). Thus, risk prediction plays a central role in the field of cardiovascular disease prevention, notably in a subgroup predicted to be at intermediate risk by traditional models, for which the consideration of new risk markers can help reclassify some individuals and, consequently, may influence clinical decision making (11, 13–15).

In this context, it is essential to remember that the most commonly found cause of CAD is atherosclerosis (16) and, for decades, the traditional view that the formation of an atheroma has followed an inexorably progressive course with age playing a prominent role in disease analysis. Over the past few years, increased evidence has pointed to the role of inflammation in the atheroma development, which has forced many of us to rethink our classical views of atherosclerosis as a segmental or localized disease (17, 18).

Coronary sudden occlusion is often preceded by a variable period of plaque instability and thrombus evolution before the onset of symptoms (19) and it occurs at sites of angiographically mild coronary-artery stenosis, as showed in the PROSPECT trial (20), which confirmed the hypothesis that ACS arise from atheromas with certain histopathological characteristics, and these characteristics are not necessarily dependent on the degree of angiographic stenosis at that particular site.

Similarly, other studies (21, 22) also concluded that thrombotic complications do not always arise at the sites where the most severe arterial narrowing by plaques occurs and coronary events are mostly due to acute thrombosis after erosion of an unstable non-obstructive atherosclerotic plaque leading to downstream ischemic events. In the CONFIRM registry, 34% of patients had only non-obstructive lesions (23). Kramer et al. (19) evaluated the relationship between thrombus healing and underlying plaque morphology in sudden coronary death and found that non-critical stenosis was apparent in at least 40% of lesions where 60% were erosions showing greater maturation of thrombi. These data further support the finding that thrombus initiation, in a substantial number of cases, occurs before the onset of symptomatic coronary events. However, plaques are very heterogeneous in size and composition, even plaques located next to each other and exposed to the same systemic risk factors. Thus, emerging concepts of the mechanisms of plaque erosion are focused on the so-called “vulnerable plaque” (20, 24–26).

Given that research on the molecular basis of atherosclerosis has made considerable inroads into understanding the pathophysiological basis of plaque rupture and has refined the understanding of this disease, it has also forced us to review the best strategies to prevent it. This discussion is timely because not all coronary events occur in individuals with traditional multiple risk factors. In some individuals, abnormalities linked to the inflammatory process, hemostasis, and/or thrombosis alone seem to play decisive roles. At the time of the first infarction, more than 75% of patients are not under preventive measures based on probability scores of traditional CV events (27). Risk estimation is inaccurate (28) and relies on group averages whose results are then applied to individual patients, often leading to the patient misclassification, which may ultimately subject the individual to undertreatment or overtreatment (29). Patel et al. (30) showed that only 41% of patients who undergo invasive coronary angiography for diagnostic purposes actually have obstructive CAD–a decidedly low proportion considering both adverse events and radiation exposure associated with invasive coronary angiography. Additionally, Pen et al. (31) investigated the association of Framingham Risk Score, a classic risk stratification tool for CAD, with coronary computed tomography angiography (CCTA) measures of coronary atherosclerosis. CCTA identified atherosclerosis in a significant proportion of patients with low to intermediate risk score, exposing a discordance between clinical score and atherosclerotic plaque burden.

**Figure 1:**
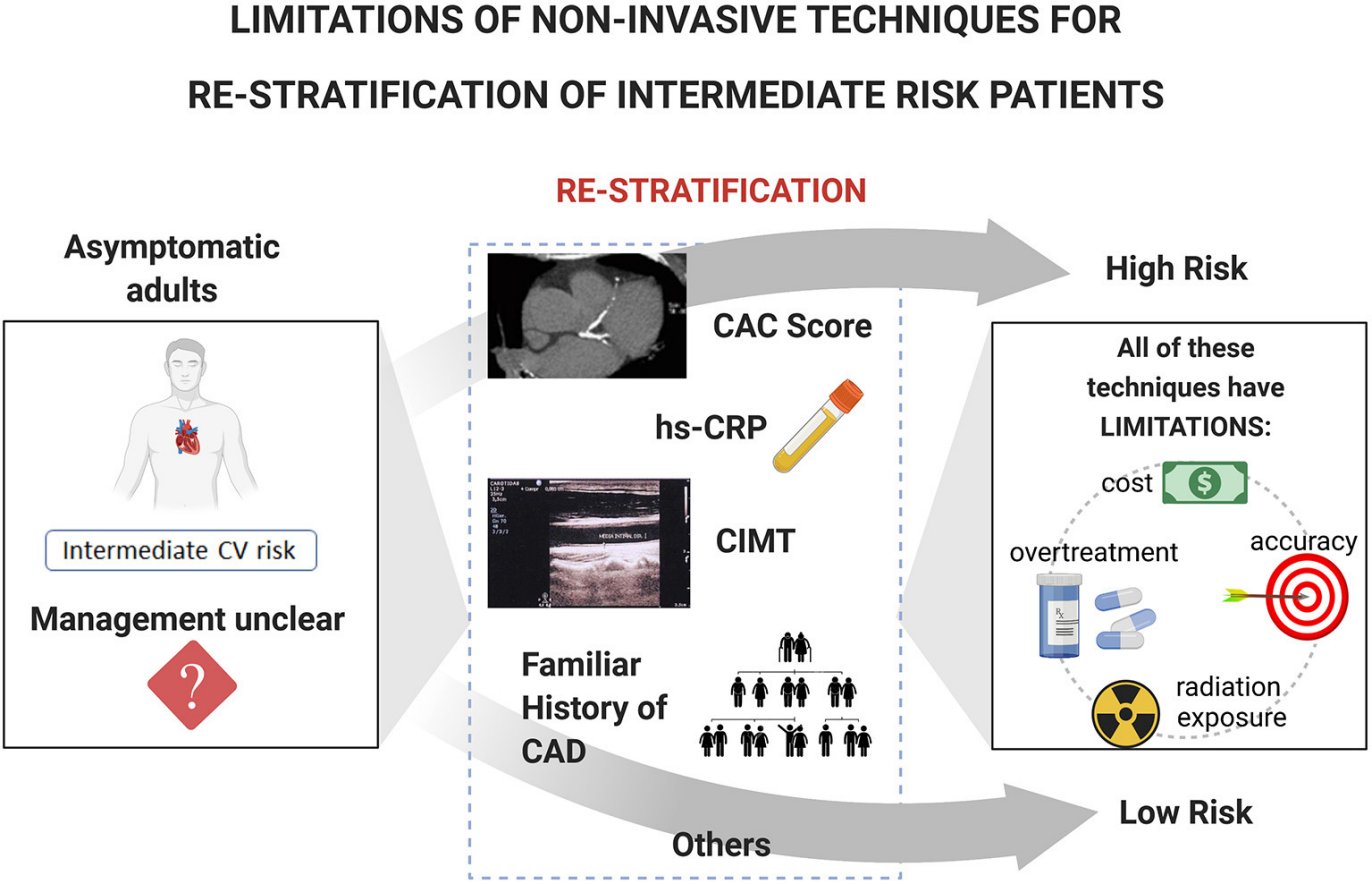
Limitations of current non-invasive techniques for re-stratification of intermediate risk patients. The management of asymptomatic subjects with intermediate risk is considered uncertain and challenger. The ability to re-stratify these patients as either low or high risk would confer important benefits. Limitations of the non-invasive techniques considered as risk modifiers to improve risk prediction and decision making include cost, accuracy, overtreatment, and radiation exposure. CV, Cardiovascular; CAC, Coronary Artery Calcium score; hs-CRP, high-sensitivity C-Reactive Protein; CIMT, Carotid Intima-Media Thickness; CAD, Coronary Artery Disease.

Ideally, a physician should be able to accurately assess the absolute CV risk of an individual patient, to calculate the likelihood of benefit or impairment of an intervention, and to prescribe therapies after a discussion of specific patient risks and benefits (32). Current trends in primary prevention advocate the need to understand cardiovascular disease (CVD) risk assessment as a process, not as a calculation. Therefore, three steps are suggested: to estimate, to personalize risk, and to reclassify the patient if necessary. Using previous guidelines, a large intermediate risk group [6–20 or 10–20% 10-year risk of developing CAD) has been identified (33)]. Greenland et al. (12) analyzed the North American adult population and estimated that 35% of individuals are in the low-risk group, 40% in the intermediate-risk group, and 25% in the high-risk group. The association of Framingham Risk Score (FRS) with obstructive CAD and proximal atherosclerotic plaque was tested by Nair et al. (34): in the low- and intermediate-FRS group, a significant proportion of individuals had proximal atherosclerotic plaque (75%) or obstructive CAD (34%), although many were not assigned statin therapy. Actually, the intermediate risk group encompassed various subjects who did not fit within the same “big box” classification. Despite the prevalence, treatment decisions were not well-defined for these patients. To re-stratify them, an array of non-invasive techniques may be considered as risk modifiers to improve risk prediction and decision making, including serum biomarkers and imaging tests (35–38). However, all of these techniques have limitations and restrains to consider (Figure 1).

Developing novel approaches to CAD risk stratification that allow more appropriate and effective primary prevention management strategies is both challenging and necessary. In this context, advances in omic techniques have provided a framework for the development of clinically useful tools for subclinical CVD diagnosis that will lead to an improved reliability of cardiovascular risk prediction beyond conventional risk factor score. More recently, metabolomics has emerged as a means of evaluating comparatively chemical intermediates, or metabolites, in a variety of biological samples, at pre-established conditions. Metabolomic research has considerable potential for translating the information comprised in the metabolic fingerprint into personalized therapeutic strategies (22, 39–41).

In order to gain a more comprehensive understanding of the pathophysiology of atherosclerosis at the molecular level, several studies have shown an association between certain metabolites and CAD (42–45). CVD has been studied by metabolomics and/or lipidomics to enhance our knowledge of molecular mechanisms associated with several heart pathologies (46, 47) and treatments (48), to prospect new drugs (49), to validate new diagnostics (50–53) and prognostics strategies (52), as well to establish novel risk biomarkers. In some of the studies focused on risk biomarker searching (43, 44), the implicated metabolites were independently associated with CAD even after adjustment for traditional CVD risk factors and were found to have incremental value for discrimination of individuals with CAD relative to the common factors. More recently, much larger combined population-based cohorts were screened by metabolomic and/or lipidomic strategies in the search of risk factors biomarkers; such endeavors can improve risk assessment reliability and help to translate the findings directly into clinical applications in a much expedite manner (54–57).

**Figure 2:**
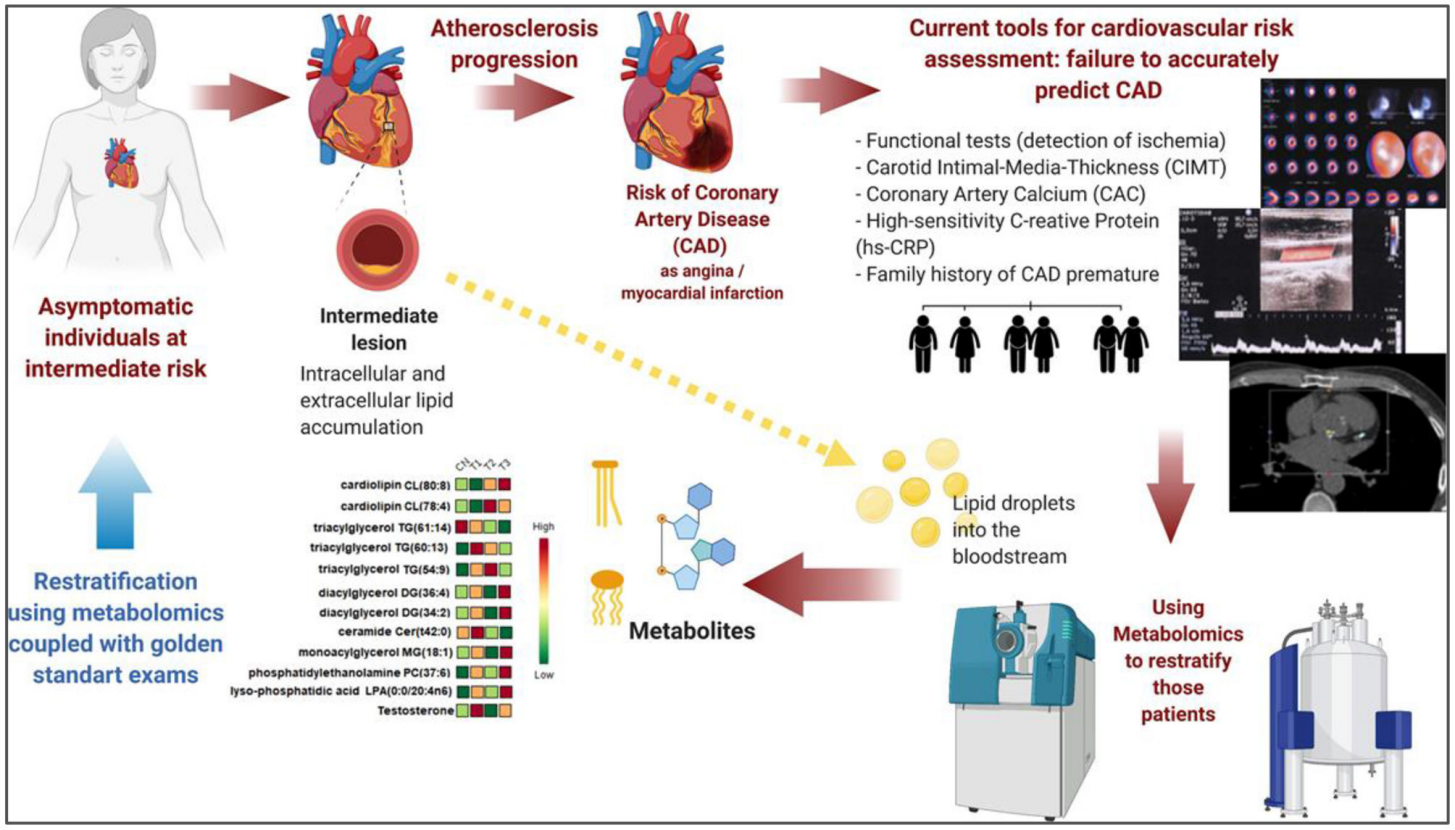
Metabolomics approaches in CAD risk stratification. Inaccurate stratification using current models is a challenge to be overcome, particularly in the group of asymptomatic individuals at intermediate risk for CAD. Current tools for cardiovascular risk assessment usually fail to accurately predict CAD asymptomatic subjects. Discriminant metabolites originated from metabolomics approaches may become promising candidate molecules to aid CAD risk stratification. Prospective studies with metabolomic's biomarkers usually apply MS instruments and/or NMR as main analytical techniques. Once the effectors from the plaques (possible lipid droplets and/or exosomes) that are carried in plasma are extracted and injected in those analytical instruments. Abundant metabolite ions are detected and identified after data processing and chemometrics approach. This molecular signature could be integrated to clinical and laboratorial data to restratify intermediate subjects. CAD, Coronary Artery Disease; CIMT, Carotid Intima-Media Thickness; CAC, Coronary Artery Calcium score; hs-CRP, high-sensitivity C-Reactive Protein; MS, mass spectrometry; NMR, nuclear magnetic resonance spectroscopy.

In this Review article, we discuss a few insights on the nature of CAD, in a historical perspective, highlighting the clinical and imaging assessments of current models for cardiovascular risk as well their limitations. It is worth noting that, since we are in the post-genomic era, where biological studies are characterized by the rapid development and wide application portfolio of multiomic technologies, we specifically focus our revision on the use of mass spectrometry and nuclear magnetic resonance spectroscopy as an analytical platform for metabolomics and its contributory role for the primary prevention of CAD (Figure 2). Although the impact and scope of molecular based risk prevention schemes toward “personalized risk assessment” are not entirely clear yet, we expect that this Review article will provide clinicians with informed knowledge about the potential benefits of this complementary strategy in cardiovascular prevention.

## Atherogenesis and CAD

The understanding of the atherogenesis mechanisms has dramatically evolved over the past 30 years. The role of cholesterol in atherogenesis has long been reported, but only in the last decade of the 20th century, strong scientific evidence started pointing to lipid deposition in the arterial wall as just the ignition point of atherosclerosis, from which a complex myriad of inflammatory events succeeds (16, 58–61).

Atherosclerosis consists in the development of plaques in any arterial bed segment, underlying most cases of ischemic heart disease, ischemic strokes, and peripheral vascular disease. Once formed, the atheroma may progress and increase enough to become an ischemic flow-limiting lesion. Alternatively, atherosclerotic plaques may also follow another path based mostly on erosion or rupture, ultimately complicated by thrombus formation, occlusion of the vessel lumen and acute tissue ischemia. The distinct trajectories of the atheroma may result from several factors, ranging from plaque microenvironment and composition to the presence and magnitude of systemic traditional and non-traditional risk factors.

**Figure 3:**
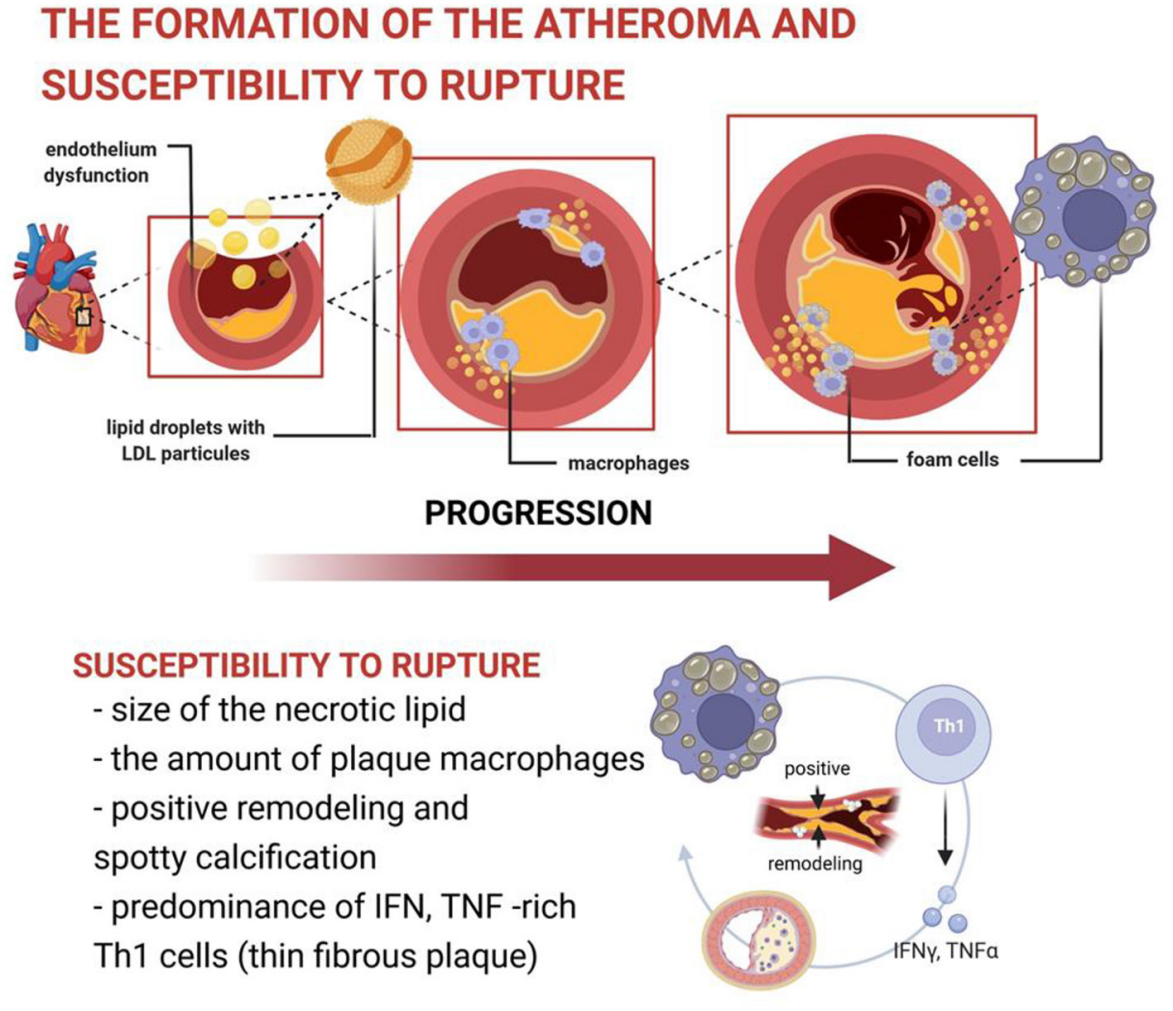
The formation of the atheroma and susceptibility to rupture. The formation of the atheroma derives from an insidious sequence of events starting with entry and accumulation of LDL particles within the sub endothelial space, more specifically in the intima. Once trapped by molecules of the extracellular matrix, those particles are more susceptible to biochemical modifications, including oxidation, which turn them pro-inflammatory. While LDL can accumulate in the intima, a dysfunctional endothelium facilitates the entry of circulating inflammatory cells. Indeed, the exposure of the endothelial monolayer to risk factors unbalances several of its properties, resulting in reduced production of endogenous vasodilators, and expression of adhesion molecules and chemo attractants, which lead to inflammatory cell accumulation in the embryonary atheroma. Distinct inflammatory cells can participate in atherogenesis. Macrophages can internalize local accumulated lipids and become foam cells. Upon cell death, lipids and debris from dead cells can form the atheroma lipid necrotic core. The susceptibility of the plaque to rupture depends on the size of the necrotic lipid, the amount of plaque macrophages, presence of positive remodeling, spotty calcification, and predominance of IFN, TNF-rich Th1 cells (thin fibrous plaque). LDL, low-density lipoprotein cholesterol; IFN, interferon; TNF, tumor necrosis factor; Th1: Type 1 T helper.

The formation of the atheroma derives from an insidious sequence of events starting with entry and accumulation of low-density lipoprotein (LDL) particles as well as cholesterol-rich remnants of VLDL within the sub endothelial space, more specifically in the intima (16, 61), as shown in Figure 3. Distinct inflammatory cells can participate in atherogenesis, particularly macrophages and lymphocytes.

The immune responses underlying atherosclerosis development and progression might be modulated by the interaction between T cells and circulating lipids. Reilly et al. discussed the importance of understanding the effects of fatty acids (FAs) on T cells. By interactions with specific FAs in the circulation, these cells undergo metabolic and functional changes, including notably its activation, proliferation, and polarization. This so-called metabolic reprogramming might be linked to the outcome of atherosclerosis (62).

Indeed, T cells, as important orchestrators of local inflammation, may induce distinct effects, either pro- or anti-inflammatory, depending on their secreted cytokines. Importantly, the synthesis of extracellular matrix molecules is also influenced by those different cytokines. The Th1 pro-inflammatory cytokine interferon-γ (IFN-γ) reduces the ability of smooth muscle cells to produce interstitial collagen, a critical component of the fibrous cap that lies over the necrotic lipid core. On the other hand, the T regulatory cell cytokine transforming growth factor-β (TGF-β) can act in the opposite way, promoting collagen synthesis. Therefore, atherosclerotic plaques that are characterized by predominance of IFN-γ-producing Th1 cells may be more prone to rupture, given their thinner fibrous cap. Besides collagen synthesis, the magnitude of collagen breakdown induced by inflammatory cells collagenases also interferes with fibrous cap susceptibility to rupture and thus plaque vulnerability. As a result, an impaired collagen synthesis and enhanced collagen degradation, both inflammation-induced phenomena, are important contributors to fibrous cap rupture. In addition to fibrous cap thickness, several other plaque features also govern plaque susceptibility to rupture, including size of the necrotic lipid core, amount of plaque macrophages, presence of plaque positive remodeling, and spotty calcification (Figure 3).

Plaque rupture is the most important mechanism underlying acute thrombosis of coronary arteries. As soon as the fibrous cap ruptures, exposure of the atheroma content to luminal blood triggers thrombus formation, ultimately culminating in significant obstruction to the coronary flow, and acute ischemic coronary syndromes. Plaque erosion is another mechanism of acute thrombotic complications of atherosclerosis, characterized by the formation of a thrombus in a region of endothelial denudation without rupture of fibrous cap (63). Attenuation of inflammation and lipid accumulation in the atheroma due to more effective therapies against atherosclerosis is probably one of the explanations for the increasing proportion of plaque erosion-derived acute coronary syndromes (16, 63).

Not all atherosclerotic plaques undergo thrombotic complications, but they can still progress and become flow-limiting atheroma. Under increased myocardial oxygen demands, such lesions impede appropriate supply of oxygen to the myocardium, leading to ischemia.

A breakthrough in the comprehension of the molecular mechanisms governing the atherosclerosis pathology derived from metabolomic studies that attempted an association of the gut microbiome metabolism with atherosclerosis development (64). Recent studies in animals have shown a mechanistic link between intestinal microbial metabolism of choline and CAD, justifying further studies testing dietary phosphatidylcholine in humans (65). The impacting work of Wang et al. demonstrated that choline, trimethylamine N-oxide (TMAO), and betaine were discriminant metabolites for CVD risk (66). The study was validated by an independent cohort (*n* = 1,876). The authors also investigated mouse models to establish the connection between dietary choline and gut microbiota during TMAO production, which resulted in an increase in macrophage, foam cell formation and cholesterol accumulation. According to Qi et al., meta-analysis of data from 11 cohort studies linking TMAO plasma/serum levels with increased CVD risk also confirmed the value of TMAO as a prognostic biomarker (67). However, Griffin et al. reviewed the evidences of gut microbiota-host interactions related to cardiovascular diseases and pointed out that care must be taken to translating the use of gut microbiota metabolites, such as TMAO, to clinical use, due to the fact that the gut microbiome composition and activity may be altered by many factors, especially diet (68).

Despite the broad and growing understanding about atherosclerosis pathogenesis, there is still a considerable knowledge gap regarding the mechanisms and triggers of plaque complications, particularly the thrombotic outcomes. A deeper comprehension of all the factors, intra and extra-plaque, underlying acute thrombosis, and improved prediction and detection of the so-called vulnerable plaques still warrant further studies.

## Current Risk Assessment for CAD

### Clinical Scores by Traditional Risk Factors

Cardiovascular risk assessment is the first and critical step in the current approach to the primary prevention of atherosclerotic cardiovascular disease (15) based on scores that estimate the risk of CVD over 10 years ahead, in order to facilitate clinical decision making (5). There are several CVD risk calculators in widespread use. The field is dynamic, with new algorithms being developed on a regular basis, which are adopted by regional organizations and societies. Providing a platform for shared decision-making between physician and patient (5, 8, 32), scores must be easy to apply with high accuracy at the patient's bedside. A general concern in screening is its potential to do harm. False positive results can cause unnecessary concern and medical treatment. Conversely, false negative results may lead to inappropriate reassurance and a lack of lifestyle changes (32).

The Framingham Heart Study (FHS) (69) pioneered the prediction of the population at risk of atherothrombotic disease and still remains the basis upon which the current predictive tools are based. According to the current guidelines, the patients should be classified in a specific group: Low Risk (<5%), Borderline Risk (5 to <7.5%), Intermediate Risk (7.5 to <20%), or High Risk (≥20%) (70). Examples of clinical scores as Framingham Risk Score (FRS), Pooled Cohort Equations (PCE), SCORE2/SCORE2-OP and Reynolds Score are shown in Table 1. The different risk models are appropriate for most individuals of the general population. Nonetheless, it must be taken into account that risk prediction should be done with a model that has been developed and validated in the population of interest because they all have limitations.

**Table 1:** Characteristics of the current clinical scores by traditional risk factor.

**cvD risk estimation scores**	**Variables**	**Recommended by guidelines**	**Limitations**	**References**
Framingham Risk Score (FRS)	Sex, age, total cholesterol, HDL-C, SBP, current smoking, hypertensive therapy, and DM.	NCEP guidelines, Canadian CV guidelines, and other national guidelines recommend adapted versions including New Zealand.	• “Mismatch” between the predicted risk and the actual plaque burden. • Validated with sex-specific equations for white, so its utility to other racial/ethnic groups is unclear. • Limited age range (30–75 years).	(15, 31, 34, 71–75)
PCE (Pooled Cohort Equations)	Sex, age, race, total cholesterol, HDL-C, SBP, antihypertensive treatment, DM, and smoking status.	2019 AHA/ACC Guideline on the assessment of CVD risk.	• May overestimate risk in groups with predicted 10-year risk >10% or higher socioeconomic status, or those receiving consistent screening and preventive care. • Tends to underestimate patients with lower socioeconomic status or with chronic inflammatory diseases	(1, 15, 70, 76, 77)
SCORE2 (systematic coronary risk evaluation)/SCORE2-OP	Sex-specific and competing risk-adjusted models, including age, smoking status, systolic blood pressure, and total- and HDL-cholesterol.	2021 European Guidelines on CVD Prevention.	• Not was evaluated in non-European populations: its value in such settings is not entirely known. • Not was compared its performance with other risk equations already developed for use in specific high-income countries. • Limited age range (40–69 years) ^*^For individuals over the age of 70, a separate risk score, SCORE2-OP, has been derived and published.	(78, 79)
Reynolds Score	Sex, age, SBP, smoking, hsCRP, total cholesterol, HDL-C, family history of premature MI, and HbA1c if diabetic.	2019 AHA/ACC Guideline on the assessment of CVD risk. Recommended in a population with characteristics similar to those of the evaluated patient.	For including new risk factors, the score becomes more complex, time consuming, and costly.	(71, 80–82)

*CVD, Cardiovascular Disease; HDL-C, High-density Lipoprotein Cholesterol; SBP, Systolic Blood Pressure; DM, Diabetes; hsCRP, high-sensitivity C-Reactive Protein; MI, Myocardial Infarction; HbA1c, hemoglobin A1c; NCEP, National Cholesterol Education Program; PCE, Pooled Cohort Equations; ACC, American College of Cardiology; AHA, American Heart Association; SCORE2, Systematic Coronary Risk Estimation2; SCORE2-OP, Systematic Coronary Risk Estimation2-Older Persons*.

It is worth mentioning here that the numerical information gathered by metabolomic studies, whether a given discriminant metabolite has increased or decreased, and by what extent, can be organized to compose new scores and/or used in conjunction with existent risk factor models to enhance their prediction reliability. The work of McGranaghan et al. (83) is a fair example of such conduct. The authors evaluated the predictive value of metabolomic biomarkers for CVD risk. They performed a meta-analysis on the results from 22 select studies regarding clinical initiatives with restrict inclusion/exclusion criteria. Details of this that review article are better discussed in the section on *Metabolomics in the assessment of cardiovascular risk*.

### Direct Measures of Arterial Structures

Not only the morphology of the individual coronary plaque, but also the total atherosclerotic burden is important for risk prediction. CAC score, which is a non-contrast enhanced ECG-gated computed tomography (CT), was the first non-invasive technique able to evaluate coronary atherosclerotic burden. Despite CAC only depicting calcified plaques, there is a good correlation between calcified plaque burden and total atherosclerotic burden (84). It is an established tool for screening asymptomatic patients and predicting ACS and death (14, 84), better predicts CV events and offers higher incremental prognostic value over clinical scores, compared to other risk markers (14).

The first CAC score algorithm was proposed in 1990 by Agatston et al. (85). Conceptually, the Agatston score is a summed score of all coronary calcified lesions, accounting for both the total area and the maximal density of coronary calcification. Easily obtainable and validated in a wide range of studies and populations (86–90), the Agatston protocol remains the most frequently applied method in clinical practice despite the discussion around the ideal scoring algorithm and the possible need for an updated CAC score (89, 90).

For a significant percentage of at-risk individuals stratified in the borderline or ambiguous categories, CAC score demonstrated to improve risk prediction with enhanced correlation between advancing CAC scores and risk progression (86, 91–93). In fact, the concept of negative risk attributed to CAC = 0 is now a consensus for the stipulation of patients unlikely to benefit from an intense statin treatment and thus off the path to proficiency in primary prevention (70, 93–95).

The contribution of metabolomics to the characterization of plaque structure relies on studies that use microscopic imaging mass spectrometry to reveal metabolic signatures throughout specific plaque tissue and/or intima tissue locations (96). The so-called metabolic phenotyping of atherosclerotic plaques in comparison to control vessels has revealed latent associations between free cholesterol and ceramide metabolism during atherogenesis (97). Moreover, metabolites in the purine and glutathione pathways indicate deregulation of oxidative stress in dissected plaque extracts (98). Another interesting observation is the increased level of quinic acid in plaques, a metabolite that promotes an inhibitory effect on inflammatory activation and oxidative stress in macrophages (98). These deregulated metabolites and pathways modulations may pave the way to the discovery of novel targets for therapeutic intervention.

### Stress Testing for Myocardial Ischemia

Stress testing provides a controlled environment for observing the effects of the increased myocardial demand for oxygen; significant fixed stenosis from coronary artery disease result in evidence of ischemia. This is the rationale for the fact that stress testing has been used for decades as a diagnostic tool in the work-up of patients with suspected CAD (99).

Exercise stress testing is a validated diagnostic test for CAD in symptomatic patients, and it is used in the evaluation of patients with known cardiac disease. By contrast, testing of asymptomatic patients is not recommended as a routine screening modality (100). Although non-electrocardiographic measures, including functional capacity, chronotropic response, heart rate recovery (HRR) and ventricular ectopy have been shown to predict adverse events in asymptomatic subjects, there is no evidence that gaining this knowledge improves outcomes (101).

Previous studies investigated the use of exercise testing in asymptomatic subjects without known CAD but with certain CVD risk factors. Greenland et al. recommended that all subjects undergo global risk assessment based on office tools such as the FRS (12). Subjects who are deemed to be at low risk for a cardiac event need not undergo any further evaluation, whereas those deemed to be at high risk for such events deserve to undergo aggressive treatment. There may be a role for screening in patients who are at intermediate risk of events. The authors noted 4 tests that may be of value: exercise electrocardiography, carotid ultrasound, CAC scanning, and ankle-brachial indexes.

Additionally, in a cohort study of asymptomatic individuals at low or intermediate FRS risk (102), it was evaluated whether two measures obtained from exercise treadmill testing, exercise capacity, and HRR, could provide incremental prognostic value for CV mortality in a very large number of men and women with lengthy follow-up (20.5 ± 3.6 years). The application of these two measures to individuals with FRS 6–19% may identify a significant proportion of those who are at high risk but might have been misclassified as at low or intermediate risk by FRS alone.

In a study of asymptomatic intermediate-risk patients, Galper et al. evaluated the cost-effectiveness of non-invasive stress cardiac testing to guide primary prevention (103). They tested the hypothesis that further risk stratification of intermediate-risk persons with stress testing might be more effective and less costly than other primary prevention approaches. The authors concluded that universal non-invasive cardiac stress testing to guide the use of statins or aspirin is not cost effective unless testing markedly increases medication adherence to about 75%.

A randomized controlled trial, The DIAD Study, involving 1,123 asymptomatic patients who had type 2 diabetes and no known CAD, found that screening with adenosine-stress radionuclide myocardial perfusion imaging did not reduce non-fatal MIs or cardiac deaths over 4.8 years compared with no screening (104).

More recently, Bauters and Lemesle performed a systematic review and meta-analysis of randomized trials addressing that the screening of diabetic patients for the presence of asymptomatic CAD may potentially impact therapeutic management and outcome (105). The screening strategy had no detectable impact on outcome with odds ratios (OR) [95 % CI of 1.00 [0.67–1.50], 0.72 [0.33–1.57], 0.71 [0.40–1.27], and 0.60 [0.23–1.52] for all-cause death, CV death, non-fatal MI, and the composite CV death or non-fatal MI, respectively.

Because direct evidence on possible benefits of screening exercise tolerance testing is lacking, US Preventive Services Task Force published a systematic review (106) suggesting that when the risk for CV events is low, most positive findings will be false and may result in unnecessary further testing or worry.

A few pilot metabolomics studies applied stress testing to uncover metabolites anticipating heart failure conditions. Sabatine et al. studied a small cohort of 36 patients, 18 of whom demonstrated inducible ischemia (cases) and 18 of whom did not (controls) (107). They found that lactic acid and metabolites involved in skeletal muscle AMP catabolism increased in both groups. However, 6 effectors including citric acid, were among the 23 regulated metabolites described by the authors, and might be potential biomarkers in ischemic cardiac events. Limkakeng et al. built a model with amino acids and acylcarnitines levels in plasma samples drawn from 20 male subjects up to 1 h before and 2 h after stress testing to predict ischemia and achieved 65% sensitivity and 60% specificity (108). Lema et al. compared metabolic alterations between groups of 244 patients undergoing either pharmacological stress tests or exercise stress tests on a treadmill and concluded that although they were able to characterize the metabolic profile of patients at risk of CV events, the pharmacological stress test did not reproduce the same dynamic changes observed in exercise stress (109).

In summary, because few prognostic studies have included adequate numbers of asymptomatic people, data are scarce regarding the prognostic utility of stress testing for the detection of myocardial ischemia in subjects with no symptoms.

### CCTA Based Risk Assessment

Coronary computed tomography angiography (CCTA) has become a valuable non-invasive tool for reliable evaluation of CAD. This methodology is able to depict not only the vessel lumen but also the wall. Recent technological advances have enabled CCTA to help with the characterization of plaque morphology in obstructive and non-obstructive disease. Traditionally, atherosclerotic plaques have been classified as non-calcified, calcified or partly calcified (also called mixed) by CCTA (110). Investigations have indicated some plaque features that are more associated with rupture, thus raising the concept of “vulnerable” plaque (111): thin fibrous cap, lipid rich plaque with large necrotic core, positive remodeling (112), heterogeneous lesion (113), and spotty or microcalcification within the plaque (114). Although CCTA cannot provide information about all of these aspects due to spatial resolution limits (as fibrous cap thickness and microcalcification), some of them are actually well-assessed by the exam (112–114).

Plaque core and positive remodeling can be evaluated by CCTA. In large necrotic core plaques, it is usually possible to measure attenuation value in Hounsfield units (HU). Low attenuation cores (<30 HU) seen on CT have good correlation with IVUS proven lipid rich plaques (115). A remodeling index was suggested to quantify positive remodeling: it is calculated as the vessel cross-sectional area at the site of maximal stenosis divided by the average of proximal and distal reference cross-sectional areas (116). A remodeling index ≥1.1 is assumed as the threshold of positive remodeling in CCTA. Low-attenuation and positive remodeling plaques were associated with acute coronary events (112).

Plaque heterogeneity may be recognized by CCTA based on its attenuation patterns. The so-called “napkin-ring sign” is also a marker of plaque heterogeneity. It is described as a plaque center of low attenuation adjacent to the lumen and a ring-like higher attenuation annular pattern surrounding the core (113). This sign was found to be an independent predictor of acute coronary syndrome (ACS) (117). Finally, spotty calcification (defined as <3 mm calcified nodules within the plaque, surrounded by non-calcified components) can be easily depicted by CCTA and it was associated with higher occurrence of ACS (114). Thus, calcification within a plaque should not be assumed as a sign of “stability.”

CCTA has emerged as a more complete tool for coronary risk stratification, since it permits evaluation of both calcified and non-calcified plaques, as well as luminal stenosis grading. Current guidelines do not routinely recommend contrast-enhanced ECG-gated CT as a screening method of asymptomatic patients (5, 118). But future improvements in radiation dose reduction strategies, as well as the use of less amount of iodinated contrast, may change such scenario. The following plaque features seem to be associated with cardiovascular events: composition (as mentioned above, large lipid rich thin cap plaques and heterogeneous plaques are more predictive of ACS); severity (stenosis grade is also an independent prognostic marker); location (plaques arising on main coronary arteries, like left main coronary artery or proximal left anterior descending coronary, carry a worse prognosis); and extent (number of coronary segments involved by atherosclerosis).

Several scores were developed in order to quantify atherosclerotic burden by CCTA but most of them do not include all those plaque features: the segment involvement score (SIS) uses the total number of segments with plaque, obstructive or non-obstructive (119); the segment stenosis score (SSS); and the CAD-RADS are based on degree of stenosis (119, 120). On the other hand, the Adapted Leaman Score (121) and, more recently, the Comprehensive CTA Score (122) assess all those plaque characteristics. Leaman Score is well-validated as a strong prognostic marker and demonstrated better cardiovascular event prediction when compared to SIS and SSS (123, 124). In a recent study, the Comprehensive CTA Score was superior to the CADS-RAD in predicting ACS (122).

## Intermediate-Risk: Practical Approach

### Reclassification of CV Risk

Overestimate 10-year ASCVD risk may lead to overtreatment. The opposite is also true. The first point to stand out is that this estimate is dominated by chronological age and not true biological age. Indeed, until the present moment, both anatomical and functional tests are unable to achieve good accuracy for all patients in all circumstances. Because of this, a substantial body of literature has been devoted over the past years to improving the prediction of CAD beyond the traditional risk score.

In addition to providing a significant independent risk rate associated with the incidence of CVD beyond what is already known based on traditional risk factors, a marker must be able to carry discrimination and reclassification power. Reclassification assesses the proportion of individuals adequately moved between risk categories by the application of the biomarker (125). In general, it is of most value and clinical utility when the individual's risk lies close to a decisional threshold (32).

There are ongoing efforts to search novel markers that could offer greater discrimination between higher- and lower-risk patients within the intermediate-risk group, including circulating, imaging, and genetic biomarkers. Although many of these markers have already defined association with future clinical outcomes, so far they are limited in terms of capacity for discrimination, calibration and reclassification (125). As an example, when hsCRP > 3.0 mg/L was considered in a model with traditional risk factors for women classified in the middle of the predicted risk spectrum (FRS predicted risk of 5–9%) during the Women's Health Study (36), an observed event rate that was equal to or greater than that of some women with an FRS-predicted risk of >10% was registered. By contrast, other studies have found either modest (36) or absent (126, 127) improvements in model calibration with the addition of hsCRP.

Risk markers have recently been compared directly with each other, including CAC, CIMT, ABI, brachial flow-mediated dilation, and hsCRP for asymptomatic individuals classified as intermediate risk group. A direct comparison of the participants' factors showed that CAC, ABI, hsCRP, and family history were independent predictors of incident cardiac or CVD in these individuals. CAC provided superior discrimination and risk reclassification compared with other risk markers (14).

A subsequent study (35) by the same authors evaluated the predictive accuracy and improvement in reclassification gained by the addition of CAC score, ABI, hsCRP levels, and family history of ASCVD to the PCE in participants of MESA (Multi-Ethnic Study of Atherosclerosis). The authors found that CAC score is superior for improving ASCVD risk prediction and may be useful in individuals whose quantitative ASCVD risk-based treatment decision making is uncertain.

According to ESC (1), additional risk factors or types of individual information can modify calculated risk. Psychosocial stress, CAC score, and CIMT (may be considered at intermediate risk when a CAC score is not feasible), for example, were cited as risk modifiers. In contrast, the associations between BMI and waist circumference and CVD did not improve CVD risk prediction as assessed by reclassification.

The 2019 ACC/AHA Guideline on the Primary Prevention of Cardiovascular Disease (5) recommended that among adults at borderline and intermediate risk, one may consider additional individual risk-enhancing clinical factors that can be used to revise the 10-year ASCVD risk estimate (family history of premature ASCVD, chronic inflammatory disease, South Asian ancestry, a history of preeclampsia or preterm delivery, early menopause, erectile dysfunction, chronic kidney disease, metabolic syndrome, persistently elevated inflammatory markers, or elevated lipid biomarkers [persistently elevated primary hypertriglyceridemia (≥175 mg/dL, non-fasting); elevated hsCRP (≥2.0 mg/L); elevated Lipoprotein (a) ≥50 mg/dL or ≥125 nmol/L; elevated Apolipoprotein B (≥130 mg/dL); ABI (<0.9)]. If there is still uncertainty about the reliability of the risk estimate after these clinically available risk-enhancing factors have been considered, further testing to document subclinical coronary atherosclerosis is reasonable to reclassify more accurately the risk estimate upward or downward. CAC scoring has superior discrimination and risk reclassification as compared with other subclinical imaging marker or biomarkers. Thus, the absence of coronary artery calcium could reclassify a patient downward into a lower risk group in which preventive interventions (e.g., statins) could be postponed, while those with coronary artery calcium ≥100 AU or coronary artery calcium ≥75th percentile have ASCVD event rates for which initiation of statin therapy is reasonable. One concern is the prevalence of non-calcified plaque in patients with zero calcium score, in which cases clinical judgment about risk should prevail.

Images must be shown to offer useful prognostic information incremental to the clinical risk assessment. Mortensen et al. (128) tested a practical, disease-guided reclassification approach to statin allocation for primary prevention of ASCVD in asymptomatic elderly people. Following guideline-recommended formal risk assessment by ACC/AHA and atherosclerosis imaging (CAC and carotid plaque burden), statin-eligible individuals were down-classified to ineligible in the absence of atherosclerosis, and statin-ineligible individuals were up-classified to eligible if significant atherosclerosis is present. According to the authors, this principle facilitates an informed clinician-patient discussion, leading to an individualized treatment decision. Intermediate-risk individuals were up classified from optional to clear statin eligibility if CAC was ≥100. With CAC-guided reclassification, specificity for coronary heart disease events improved 22% (*p* < 0.0001) without any significant loss in sensitivity, yielding a binary net reclassification index (NRI) of 0.20 (*p* < 0.0001).

As mentioned before, discriminant metabolites originated from metabolomics approaches and validated by meta-analysis initiatives examining large cohorts may become promising candidate molecules to aid patient stratification for CV risk events. Ceramides, a family of lipidic metabolites, whose applicability to compose risk scores for cardiovascular disease derived from metabolomics/lipidomics studies (56, 57), were the first set of molecular biomarkers translated into clinical practice. Mayo Clinic, a renowned non-profit hospital system in the US, offers in its clinical exam portfolio the determination of ceramides with the purpose of classifying cardiovascular risk into 4 categories according to the score level. Details of pioneer works are described in the section on *Metabolomics in the assessment of cardiovascular risk*.

### The Limitations on Current CAD Evaluation

Undoubtedly, cigarette smoking, diabetes, hyperlipidemia, and hypertension, called as “conventional” risk factors, are independent risk factors for CAD (5, 32, 129–131) and there is strong evidence supporting their role in the pathogenesis of coronary atherosclerosis. However, current markers provide an incomplete view of an individual's risk for future cardiovascular events. Between 15 and 20% of all heart attacks and strokes in the US occur among individuals who do not smoke or suffer from hypertension or DM (131). When new factors like obesity and body mass index are added to score clinics, it may not improve the predictive power of the model, because of the high correlation with other factors already in the model (132).

The weight given to premature family history in risk stratification should be considered. It is estimated that only about 20% cases of atherosclerosis are genetically determined. We know that premature CAD has a genetic component, but a major contribution of genes acting in the absence of the conventional risk factors is unlikely, as suggested by the GENECARD Project (133). So, family history of CHD may simply represent a shared exposure to a higher prevalence of classic risk factors (133). Although it is clear that family history is an important determinant of risk, the complex interplay between genetic factors, environmental exposure and lifestyle choices often makes confident assessment of an individual's risk impossible.

Simple lipid profiling to measure plasma traditional lipid does not explain the existence of substantial numbers of patients that developed CVD despite having a normal range of plasma cholesterols (134). Among individuals without any prior cardiovascular disease or diabetes, 72.1% had admission LDL levels <130 mg/dL, which is the current LDL cholesterol target for this population (135). Also, even relatively small differences in LDL-C and VLDL-C levels are associated with changes in ASCVD event risk (136, 137). HDL-C and LDL-C levels had, therefore, low genetic and phenotypic correlations with most of the lipid species. Hua and Malinski (138) showed that of the three subclasses that comprise LDL, only one causes significant damage. Subclass B is the most susceptible to be oxidized (139) and may be a very valuable tool in the early diagnosis of atherosclerosis. Researchers from Finland (140), using MS-based shotgun lipidomics to define the fingerprints of lipid molecular species in CVD, analyzed the lipid measurements from the lipid panel vs. lipidomic analysis at the molecular level to see whether they provide the same degree of information on CV risk prediction. The molecular lipidomic data trumped the panel data by several orders of magnitude and produced detailed coverage of lipid molecular patterns in more than just cardiovascular diseases that previously were not known.

CIMT (Carotid Intima-Media Thickness) has several limitations in risk stratification. Since atherosclerosis is asymmetrically distributed across the carotid artery, selectively measuring only one angle is likely to ignore the asymmetric nature of the disease (141). Besides that, the CIMT-associated risk of cardiac events is also non-linear (142) and data on change in CIMT induced by lipid-level modifying or blood pressure lowering therapies and change risk for CV events are very limited (143). As of now, evidence supporting a role for CIMT measurement in individual patients is poor (144) and screening of asymptomatic patients with CIMT not prognostically useful, even in diabetic patients. Malik et al. (145) showed that diabetic patients with a CIMT in the fourth quartile had no significant increase in CV events (HR: 1.7, 95% CI: 0.7 to 4.3) compared to those in the first quartile. Den Ruijter et al. (146) demonstrated that the addition of common CIMT measurements to the FRS was associated with small improvement in 10-year risk prediction of first-time MI or stroke, but this improvement is unlikely to be of clinical importance. The net reclassification improvement with the addition of common CIMT was small (0.8%; 95% CI, 0.1–1.6%). For those at intermediate risk, the net reclassification improvement was 3.6% in all individuals (95% CI, 2.7–4.6%). Saedi et al. (147) found no relationship between CIMT and the SYNTAX score, an angiographic scoring system.

Even CAC score suffers from some limitations. First, there are concerns regarding costs and radiation exposure, which is particularly problematic in the screening of asymptomatic patients. Secondly, calcification develops late in the atherosclerotic process (148) and it does not necessarily reflect the current status of the plaque because the calcification may be inactive, ongoing, or incomplete (149). Thirdly, calcification does not directly cause ischemic heart disease events and ruptured culprit plaques are not necessarily calcified (20, 150). So, plaque non-calcified components are missed, which can decrease the ability to predict future events. Furthermore, there is a significant variability between acquisition protocols, reconstruction algorithms, and even different vendors, generating suboptimal score reproducibility (151). Finally, one commonly stated limitation for clinical CAC scoring is the absence of a risk calculator for integrating this information into global CV risk assessment (152).

## Metabolomics In CAD Risk Assessment

The current non-reductionist knowledge of CVD, particularly of CAD, requires investigation of many biological levels and examining the interactions between heterogeneous components. The dynamic biology that exists in biological systems can be accessed and explained by a systems approach. Highlighting exemplary studies, Joshi et al. recently published a Review describing a growing number of omic sciences that try to generate large datasets in which interpretation requires modern computational approaches (153). It is worth noting that Langley et al. undertook a multiomics analysis of human atherosclerotic plaques and identified molecular signature of symptomatic plaques: a tissue-based biomarker panel that included pro-inflammatory molecules that, when measured in plasma, outperformed traditional risk factors and plasma hsCRP levels as predictors of CVD in two independent cohorts (154).

Omic sciences are dedicated to the study of all biological molecules that are involved in the organization, function and dynamics of a cell, tissue or organism (155, 156). More precisely, genomics studies the structure, function, evolution and mapping of the genome; transcriptomics and micromics aims to understand the role of all RNA molecules produced by the genome; proteomics investigates the biochemical properties and functional roles of proteins; and metabolomics examines all primary and secondary metabolites, including lipids, and their fluxes in relation to a specific biologic state (156–160). As metabolites are the end products of all cellular regulatory processes, they capture a unique aspect of cellular homeostasis providing precious information about an organism's phenotype that reflects the integrated effects of genomic, transcriptomic, and proteomic variations (159, 161, 162). The two relevant analytical platforms, in extensive use for metabolomics studies, are based on nuclear magnetic resonance spectroscopy (NMR) (161, 162) and mass spectrometry (MS) hyphenated to separation techniques (163), such as gas (GC-MS) and liquid-chromatography (LC-MS), using a variety of stationary phases to encompass molecular coverage from non-polar and moderate polar metabolites (realm of the reversed-phase liquid, chromatography, RPLC-MS) to polar metabolites (realm of the hydrophilic interaction liquid chromatography, HILIC-MS), and more recently, capillary electrophoresis (CE-MS) (164), which assesses the highly polar fraction of the metabolome (162).

Mass spectrometry has a long, well-established presence in clinical laboratories, for decades now, due to important performance characteristics, such as the ability to identify accurately and to quantify with precision compounds with high analytical specificity and sensitivity, besides the capability of detecting multiple analytes of interest in a high-throughput single analysis at improved speed (165–169). Since the mid-1960s, GC-MS has become the gold standard for the analysis and quantitation of drugs, some organic acids and hormones (168, 169). In recent years, liquid chromatography coupled with tandem mass spectrometry (LC-MS/MS), using high-resolution mass analyzers such as time-of-flight, and/or quadrupole coupled to time-of-flight (TOF, QTOF), and more recently orbitraps, have gained tremendous popularity in clinical environments (165, 170, 171). Thus, LC-MS/MS has become the technique of choice at point-of-care (POCs), particularly for the analysis of hormones, proteins, drugs, and metabolites, for both screening and identification (169, 172).

With fairly recent technological advancements in the MS instrumentation, such as improved resolving power, expedite data acquisition (scan speed), remarkable mass precision and mass accuracy, fragmentation resources, among other features, it was reasonable to expect a smooth transition of MS technology into the new omic sciences domain, especially proteomics and metabolomics/lipidomics (155, 156). Mass spectrometry-based metabolomics rapidly found its niche to evaluate cardiovascular diseases (157, 172, 173).

As described elsewhere (157, 174), the exact cause and the molecular mechanisms governing atherosclerosis are unknown. Although certain conditions or habits may increase the risk for the disease, there is still a need for better classification to identify and treat patients at risk. Metabolomic studies show an unlimited potential for identifying biomarkers for better risk stratification and for improving the understanding of the pathophysiology allowing enriched diagnostic and therapeutic options for patients (173, 174).

**Figure 4:**
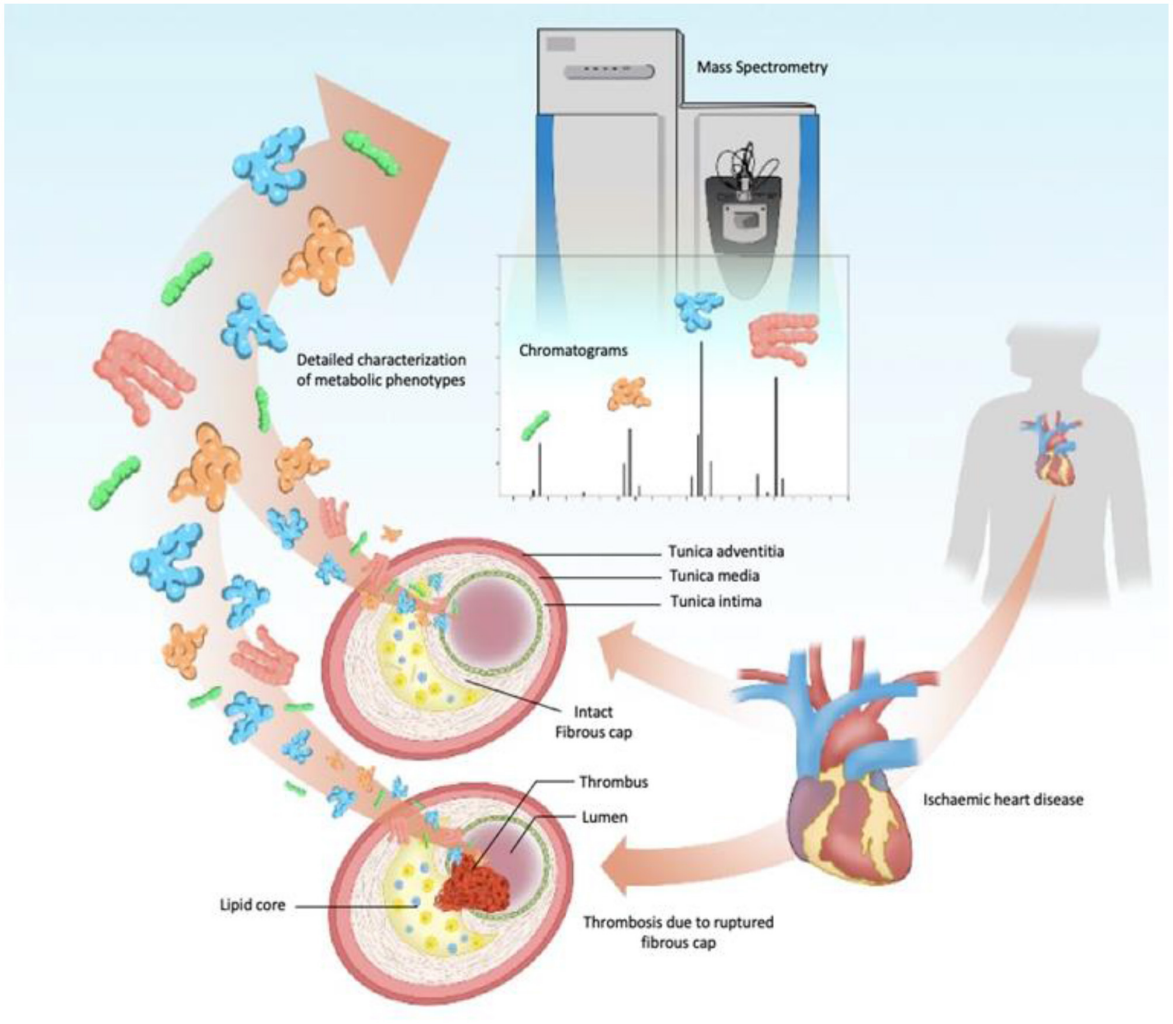
Schematic view of subclinical CAD metabolomic-signature by high-performance analytical tool. Prospective studies of metabolomic biomarkers usually apply MS instruments coupled with chromatography systems as the main analytical technique. In these protocols, once the components extracted from the plaque are injected, abundant metabolite ions are detected in specific regions of the chromatogram, and compared to a database for identification, on the mass-to-charge ratio and fragmentation patterns. Statistical analysis and network modeling complement the refinement of patients analytical data, discriminating samples from different stages and identifying metabolic pathways and biomarkers that could lead to acute coronary syndromes (angina and myocardial infarction). CAD, Coronary Artery Disease; MS, mass spectrometry.

Metabolomic screening in conjunction with the traditional risk assessments has the potential to improve non-invasive diagnostics. Metabolomics affords detailed characterization of metabolic phenotypes and can enable characterization of metabolic derangements at CAD, enabling also to quantify the vulnerability of atherosclerotic plaque to rupture (Figure 4).

Recently, McGranaghan et al. published a systematic review covering a 9-year publication span (from January/2010 to July/2019) compiling metabolomic biomarkers for CV risk (83). About 90% of the selected publications were MS-based studies showing the progress and capability of mass spectrometry in the search for metabolites as disease markers. A total of 39 biomarkers were significantly associated with fatal CVD, of which 27 were associated with higher risk and 12 with lower risk. The group of compounds containing the largest number of biomarkers reported was glycerophospholipids, with 12 different species across 6 different studies. Floegel et al. have investigated the association of acylcarnitines, amino acids, phospholipids, and hexose, with the risk of MI and ischemic stroke in two large prospective cohorts [Heidelberg and (EPIC)-Potsdam] (175). Sphingomyelins (C16:0, C24:0, and C16:1), hydroxy-sphingomyelin (C22:1), diacyl-phosphatidylcholines (C38:3 and C40:4), and acyl-alkyl-phosphatidylcholines (C36:3, C38:3, C38:4, and C40:3). They showed positive correlation with total- and LDL-cholesterol and they were associated with risk of MI in health adults at both cohorts. Even when data were additionally adjusted for total-, LDL-, and HDL-cholesterol, triglycerides, and hsCRP, C38:3, C40:4, and C36:3, glycerophospholipids metabolites remained associated with risk of MI. In contrast, no association was found between serum metabolites and risk of stroke. In summary, three metabolites involved in the arachidonic acid pathway were able to improve CVD prediction independently if traditional risk factors and other biomarkers were considered. Paynter et al. identified and validated 33 metabolites associated with CAD in postmenopausal women, 8 of each remained independently associated after adjustment for traditional risk factors: glutamine, glutamate, cytidine monophosphate, hydroxy-PCs (C34:2 and C36:4) and oxidized derivatives from arachidonic acid (15-HETE, 5-HETE, and 11-HETE) in both the discovery and validation data sets (176). C34:2 hydroxy phosphatidylcholine was identified as the strongest marker being further replicated in a third data set involving men and women. Using a different approach, a non-targeted based metabolomics study, Ganna et al. also found four lipid-related metabolites (LPC 18:1, LPC 18:2, MG 18:2, and SM 28:1) associated with incident CVD events independently of main cardiovascular risk factors in 1,028 individuals with validation in 1,670 subjects (177).

Another group of compounds that must be discussed is acylcarnitines, which presented combined size effects across different studies. Rizza et al. have demonstrated that medium- and long-chain acylcarnitines (acetyl carnitine C2, C6, C8, C10, C10:1, C12, C12:1, C14, C14:1, C14:2, C16, C16:1, C18:1, and C18:2) significantly increased the prediction accuracy of the traditional FRS, suggesting that this class of metabolites is independently associated with the occurrence of subsequent cardiovascular events in elderly individuals (178). Shah et al. profiled 69 metabolites and lipids by MS in 2,023 patients undergoing cardiac catheterization (173). By using univariate and multivariate models, they demonstrated that 5 out of 13 metabolites factors were associated with mortality. Those factors included medium-chain acylcarnitines, short-chain dicarboxylacylcarnitines, long-chain dicarboxylacylcarnitines, branched-chain amino acids, and fatty acids. The authors used risk reclassification analysis to determine whether metabolite levels could help estimating the patient's risk for cardiovascular events. Their study focused on patients classified at the intermediate risk based on clinical predictors. In total, 27.5% patients were reclassified by the metabolomic model for mortality: 19% patients were reclassified to low risk, while 8.5% were correctly reclassified to a higher level of risk.

Over the past few years, metabolomic studies have evolved to gain a much broader dimension. Large prospective cohorts have been inspected and the results combined to help establishing risk for cardiovascular events in a much more effective and reliable manner. In these cohorts, patients are also accompanied for much longer periods of time. Metabolite profiling and CV event risk was assessed by Würtz et al. in a prospective study comprised of 3 population-based cohorts, the National Finnish FINRISK study (*n* = 7,256; 800 events), Southall and Brent Revisited (SABRE; *n* = 2,622; 573 events), and the British Women's Health and Heart Study (*n* = 3,563; 368 events) (54). In the targeted analysis of 68 lipids by NMR, 33 measures were associated with incident cardiovascular events after adjusting for age, sex, BP, smoking, DM, and medication. In further meta-analysis including routine lipids, 4 metabolites were associated with future cardiovascular events: higher serum phenylalanine and monounsaturated fatty acid levels were associated with increased CV risk, whereas higher omega-6 fatty acids and docosahexaenoic acid levels were associated with lower risk. A risk score incorporating these 4 biomarkers was inserted in FINRISK. Risk prediction estimates were more accurate in the 2 validation cohorts although discrimination was not enhanced. Risk classification was particularly improved for persons in the 5–10% risk range. Biomarker associations were further corroborated with MS in FINRISK (*n* = 671) and the Framingham Offspring Study (*n* = 2,289).

In the work of Delles et al., metabolites of the cohort PROSPER (179) (PROspective Study of Pravastatin in the Elderly at Risk; *n* = 5,341; 182 events; 2.7-year follow-up) were combined with metabolites discriminated in a second cohort (54) (FINRISK 1997, *n* = 7,330; 133 events; 5-year follow-sup) and revealed that phenylalanine was replicated as a predictor of incident heart failure hospitalization (55). In another study published by Sliz et al. using NMR metabolomics platform on 5,359 blood samples from PROSPER, the authors examined the effects of statin therapy and genetic inhibition of proprotein convertase subtilisin/kexin type 9 (PCSK9). The metabolomic effects of a loss-of-function PCSK9 variant and those of therapy with statins were highly similar when assessing lipid and lipoprotein subclasses, fatty acids, and polar metabolites. These results exemplify the utility of large-scale metabolomic profiling with genetics and randomized trial data to uncover potential molecular differences between related therapeutics (180).

The most promising class of metabolites uncovered by metabolomic initiatives are ceramides. Lipidomic studies with large cohorts have associated ceramides with major adverse CV events (181) and death (182). Based on the concentration level and the ratio of four ceramides species, Cer(d18:1/16:0), Cer(d18:1/18:0), Cer(d18:1/24:0), and Cer(d18:1/24:1), a risk score termed CERT1 was developed. The score classifies the patients into four risk categories: low, moderate, increased, and high risk and it is currently implemented in the clinical practice by Mayo Clinic in the US and Finland (56). Further on, Hilvo et al. developed and validated a new score to stratify CVD risk employing the same ceramides previously used but adding phosphatidylcholine species–PC (16:0/16:0), PC (16:0/22:5), and PC (16:0/22:6–to improve the score system compared to CERT1 (57). Such scores were based on data from the cohort WECAC (*n* = 3,789) and validated with patient results from the cohorts LIPID (*n* = 5,991) and KAROLA (*n* = 1,023). Ceramide-based scores for practical use in clinical settings would gain even broader applicability when high throughput and cheaper analyses were developed.

In a prospective study by Ellims et al. (22), plasma lipidomic analysis could predict the burden of non-calcified coronary plaque in 100 asymptomatic subjects at intermediate risk of CAD according to the Framingham risk score, while other contemporary markers of CAD (CIMT, brachial-arteryPWV, and hsCRP) showed no significant relationship with the amount or type of coronary artery plaque assessed by CCTA. Eighteen lipid species demonstrated significant associations with non-calcified plaque burden, but not with total plaque or calcified plaque burden. Species of each of the six lipid classes (phosphatidylcholine, phosphatidylethanolamine, phosphatidylinositol, cholesteryl ester, G_M3_ ganglioside, and diacylglycerol) all contained fatty acids from the *de novo* lipogenesis pathway (C16:0, C16:1, C18:0, C18:1), suggesting an upregulation of *de novo* lipogenesis may be associated with non-calcified plaque burden. The authors concluded that re-stratification of these patients by plasma lipid profiling may enable more appropriate and effective primary prevention management strategies.

As very well-pointed out by Tillmann (183), in a recent editorial titled “Atherosclerotic metabolites: basic science is progressing, so we need to think about clinical implications,” there is a gap between reported CVD metabolomic biomarkers and their translation into potential clinical use or preventive care in the future. Furthermore, the publications compiled in this review have shown that the application of a single biomarker may be insufficient for evaluating cardiovascular disease pathophysiology, treatment effect and prognosis, emphasizing that a combination of multiple metabolites may be more accurate in targeting risk patients. Therefore, a recognized database with identified and validated molecular features/effectors needs to be established. Collectively, biomarkers findings could maximize the development of robust panels that would be ready for clinical testing to help improving clinical diagnosis.

Since the inception of term “metabolomics” over 20 years ago, there has been a significant discussion about the benefits of this approach in cardiovascular disease and its possible role as a CV risk stratification tool. However, several current issues must be addressed and steps must be taken to make this a reality (184). These challenges include the high capital cost of equipment, requirements for a skilled labor force, lack of automation, and regulatory uncertainty (185). Besides, as an important tool for the establishment of clinical decisions, the transition from targeted to untargeted omic approaches relying on pattern recognition is a task that needs to be mastered (165).

A necessary step toward standardization of the mass spectrometry acquisition method, with better inter-laboratory reproducibility, must be pursued before its power can be harnessed toward clinical application (186). The variability of techniques, methods and data reporting across studies are relevant aspects. For instance, details about sample preparation, phase composition and descriptive preparation, instrumental parameters and calibration, and strategies for reliable data acquisition such as column conditioning in between runs, use of quality control samples, among others are often lacking or incomplete in published studies, making independent replication of results difficult.

Metabolomics data acquired from large, well-phenotyped cohorts are required to establish the matrix of correlations between metabolite levels and clinical variables known to be associated with cardiometabolic disease (41). We also need longer durations of follow-up and repeated measures of metabolites. All results derived from each study must be validated by independent groups, in different clinical populations. Practical clinical trials are an important tool to address the translational gap.

Taken all together, the challenge of testing, promising new, rapid and non-invasive modalities for early detection of subclinical atherosclerosis and consequent prevention of the manifested disease responsible for a vast majority of deaths worldwide is a reality. In fact, the incorporation of novel biomarkers has been an on-going area of research (187) and a key part of the strategic solution is to leverage the application of metabolomics data to the existing scores (39).

Ultimately, it is essential for clinicians to be able to assess CV risk rapidly and with sufficient accuracy. The right choice of biomarkers can help driving decision-making and lower the costs, and cycle-time for progression of a new tool from the bench into the clinic. At the forefront of the 21st century, additional new data are also currently being collected on data mining and artificial intelligence which will contribute to expanding our mechanistic understanding of the integrative biology. Big data analytics using artificial intelligence (machine learning, deep learning, or cognitive computing) will enable precision cardiovascular medicine and projects as a tool that will assist physicians in making better clinical decisions.

## Conclusions

Nowadays, clinical management remains uncertain and challenging, as traditional stratification methods inaccurately predict the risk of cardiovascular events in intermediate risk asymptomatic subjects. Metabolomics coupled with high performance analytical tools, as mass spectrometry (MS) and nuclear magnetic resonance spectroscopy (NMR) would improve this knowledge, providing accurate molecular signatures for subclinical atherosclerosis, to reclassify intermediate risk asymptomatic individuals. In summary, by virtue of rich and deep phenotyping and the personification in CVD risk factors, in addition to providing cost-effective solutions to a relevant clinical problem and dealing with enormous numbers of predictors and combining them in non-linear and highly interactive ways, an implementation of traditional methods coupled with metabolomics in this field allows to achieve a major goal: to improve the practice of medicine and to enable physicians to provide better patient care. Thus, emerging as an important complementary strategy in cardiovascular prevention, to help improving clinical diagnosis and decision makers at downstream services.

## Author Contributions

AM has contributed writing the session Metabolomics in CAD Risk Assessment, Perspectives, and Conclusion, with manuscript conception, final revision, and final supervision. MP has contributed writing sessions Introduction, Challenges for metabolomic screening, and Perspectives, with manuscript conception, structure and final revision. DP has contributed writing sessions Introduction, Stress Testing for Myocardial Ischemia, and Perspectives. HM and LA have contributed writing sessions Risk assessment for coronary disease: traditional risk factors and Risk assessment for coronary disease: direct measures of arterial structures. VG and RS have contributed writing session Atherogenesis and coronary disease. RO has contributed writing session Risk assessment for coronary disease: direct measures of arterial structures, and with reference management. CP, AF, MT, and AM have contributed writing session Metabolomics in CAD Risk Assessment. MT has contributed with final revision. CB has contributed with funding support and final revision. GM has contributed writing session Risk assessment for coronary disease: CCTA based risk assessment, and deep discussion about Leaman's score. FA has contributed writing sessions Intermediate-risk: practical approach and Challenges for metabolomic screening, and with final revision. All authors contributed to the article and approved the submitted version.

## Funding

AM, CP, AF, MT, and CB were supported by the AIRBUS international offset between Spain and Brazil–ACORDO 002/DCTA-COPAC/2014.

## Conflict of Interest

The authors declare that the research was conducted in the absence of any commercial or financial relationships that could be construed as a potential conflict of interest.

## Publisher's Note

All claims expressed in this article are solely those of the authors and do not necessarily represent those of their affiliated organizations, or those of the publisher, the editors and the reviewers. Any product that may be evaluated in this article, or claim that may be made by its manufacturer, is not guaranteed or endorsed by the publisher.
